# YouTube as a learning tool for four shoulder tests

**DOI:** 10.1017/S1463423618000804

**Published:** 2018-10-30

**Authors:** Heewon Lee, Asayeon Choi, Yongjun Jang, Jong In Lee

**Affiliations:** 1 Department of Rehabilitation Medicine, Seoul St. Mary’s Hospital, College of Medicine, The Catholic University of Korea, Seoul, Republic of Korea; 2 Department of Rehabilitation Medicine, Bucheon St. Mary’s Hospital, College of Medicine, The Catholic University of Korea, Seoul, Republic of Korea

**Keywords:** YouTube, e-learning, shoulder, physical examination, e-health

## Abstract

**Aim:**

To analyze the use of YouTube videos as educational tools for four physical examinations of the shoulder: the Neer, Hawkins, empty can, and drop arm tests.

**Background:**

Video-based education, which is accompanied by text-based education, can be an effective education method, especially in learning medical skills. Medical students and doctors in training often use YouTube videos to share medical education materials, and more systematic review of the reliability of these videos is required.

**Methods:**

A search of YouTube was conducted using four keywords: ‘Neer test,’ ‘Hawkins test,’ ‘empty can test,’ and ‘drop arm test.’ Two physicians reviewed each video and recorded a variety of characteristics (date uploaded, number of views, likes and dislikes, and upload source). In addition, they scored and categorized the videos into four groups: ‘very useful,’ ‘somewhat useful,’ ‘not useful,’ and ‘misleading.’ Videos containing inappropriate content were classified as ‘misleading.’ Finally, the correlations of each video’s usefulness with viewers’ preferences and the upload source were analyzed.

**Results:**

A total of 400 videos were assessed and 200 videos were adopted which yield eligible criteria. Out of 200 videos, 51 videos were very useful and 32 were misleading. Significant correlations were observed between the video’s usefulness and the uploaded source, as well as between the video’s usefulness and viewers’ preferences, such as the number of views, views per day, and number of likes. The proportion of videos classified as ‘very useful’ was highest (58.6%) among those uploaded by physicians and lowest (12.7%) among those uploaded by individuals. Videos uploaded by individuals had significantly lower values reflecting viewer preferences than did videos uploaded by physicians.

**Conclusion:**

YouTube videos could be used as learning sources for shoulder physical examinations after the application of appropriate filtering processes, such as review of the upload source and viewers’ preferences.

## Introduction

Through the development of the worldwide web, the internet has become the largest and the most up-to-date reservoir of medical information (Choules, 2007). By taking advantage of its accessibility, e-learning has become an increasingly attractive method of medical education (Pusponegoro *et al*., 2015). Thus, many medical students and doctors now use the internet as a learning tool (Muhammed *et al*., 2014).

Owing to the simplicity of providing online content, a large number of medical multimedia materials are available on the internet in a variety of formats. As virtual simulation is used before performing actual procedures on real patients, videos with three-dimensional (3D) images and audio are potentially excellent educational aids for manual procedures, such as physical examinations. Several previous authors have argued that video-based education improves learning outcomes among medical students. In a randomized, controlled, assessor-blinded trial, video instruction group of medical students significantly improved performance of venepuncture as measured by checklist score than non-video instruction group, with scores of 14.15 and 9.18, respectively, out of a total of 18 points (Pan *et al*., 2014).

There are several open-access platforms, and YouTube is the second most popular website in the world following Google (www.google.com), accounting for 60% of all videos available online. Although similar websites such as Yahoo Video, MetaCafe, DropShots, and others are available for video sharing, the most popular video-hosting website is YouTube (www.youtube.com) (Azer *et al*., 2012) and over 4 billion videos are watched around the world every day and more than 65 000 new videos are uploaded every day (Azer *et al*., 2013). The true merit of this website is that it can be used to share medical education materials for free and can be accessed worldwide by medical students and doctors in training. However, YouTube is a consumer-generated website that is unregulated and thus carries the risk of disseminating inappropriate information.

Previous studies assessed the quality of YouTube videos as learning tools for electrocardiography (Akgun *et al*., 2014), respiratory auscultation (Sunderland *et al*., 2014), male urethral catheterization (Nason *et al*., 2015), tonic-clonic seizures (Muhammed *et al*., 2014), lumbar puncture, and neuroaxial block techniques (Rössler *et al*., 2012). Owing to the diverse quality of the content, they have been considered inadequate for educational use.

As physical examination is a procedure comprising movement in three dimensions, video examples can be extremely useful educational aids. Therefore, good-quality YouTube videos can be useful learning tools, with the added benefits of good accessibility and being cost-free. However, no study to-date has evaluated the validity of YouTube videos for shoulder physical examinations. This study aimed to investigate the usefulness of educational videos of shoulder physical examinations.

Numerous shoulder physical examinations are used to diagnose shoulder disorders. As performing all of them in each patient is not feasible, physical examination is often performed selectively. According to previous studies, rotator cuff injuries lead to a high prevalence of chronic shoulder disorders, which comprise 10% of all shoulder-related diseases, and the supraspinatus muscle is most commonly involved in rotator cuff tears. Shoulder impingement syndrome is also common, with five in 1000 individuals diagnosed as new clinical cases each year (Joo *et al*., 2017). In this study, we selected four shoulder tests for each of the rotator cuff injuries and impingement syndromes. Other studies have shown that shoulder physical examinations have high sensitivity and are useful in clinical practice (Beaudreuil *et al*., 2009; Lange *et al*., 2017).

The purpose of the present study was to assess the quality of YouTube videos as educational tools for four well-known physical examinations of the shoulder: the Neer, Hawkins, empty can, and drop arm tests.

## Materials and methods

The Institutional Review Board of the Catholic University of Korea approved this study and exempted it from ethical review.

### Search strategy

We selected four specific examinations of the shoulder: the Neer, Hawkins, empty can, and drop arm tests. Various search terms can be derived to find YouTube clips of these examinations. For example, searches for the Neer test can be performed using keywords such as ‘Neer’s sign test,’ ‘Neer impingement test,’ and ‘Neer test.’ Using Google Trends (http://www.google.com/trends/), we selected the following keywords, which were used most frequently to search for the physical examinations: ‘Neer test,’ ‘Hawkins test,’ ‘empty can test,’ and ‘drop arm test.’

After keyword selection, YouTube searches were conducted in March 2015 (‘Neer test,’ March 1, 2015; ‘Hawkins test,’ March 10, 2015; ‘empty can test’ and ‘drop arm test,’ March 11, 2015). The only search filter used was ‘relevance,’ which is the default filter for a normal YouTube search.

Using methods described previously (Nason *et al*., 2015), and with the baseline assumption that no user would go beyond the first five pages of results (20 videos per page) for each search term, videos on the first five pages were screened.

### Inclusion and exclusion criteria

Only English-language videos were included in the search. Irrelevant videos and still images were excluded. Videos of sufficiently poor quality to prevent evaluation were also excluded. The same video reposted by multiple users was treated as a single video and evaluated once. For videos with content related to multiple shoulder examinations, only the sections of interest were evaluated.

### Data assessment and review

Two physicians independently evaluated the videos and recorded characteristics including the date uploaded, the uploader, and the numbers of views, likes, and dislikes. Using these data, we calculated the number of days for which each video had been posted and the number of views per day. The upload sources were divided into three groups according to uploaders’ credentials: physicians, medical websites, and individuals. ‘Physician’ referred to an uploader based in an official hospital or professional organization, ‘medical website’ referred to an upload from a medical practitioner or unofficial hospital data, and ‘individual’ referred to an uploader of unknown credential (Kumar *et al*., 2014).

No standardized tool is available for the assessment of the quality of diagnostic information for shoulder examinations. To evaluate the quality of the videos, the authors scored the videos for each examination (Table 1), based on a review of the literature (Hermans *et al*., 2013; Jain *et al*., 2013) and previous evaluations of YouTube videos (Akgun *et al*., 2014; Lee *et al*., 2014; MacLeod *et al*., 2015). The scoring system had four components, each with a total possible score of 8: purpose, performance, positive sign, and mechanism. The scoring system contained checklists for each category, and the total number of points was calculated simply by summing the checked criteria. The maximum possible score was 8, and the minimum was 0. These scores were used to categorize videos into three groups: ‘very useful’ (6–8), ‘somewhat useful’ (3–5), and ‘not useful’ (0–2). Regardless of the score, videos containing incorrect information were classified as ‘misleading.’

**Table 1:** Customized scoring scheme

	Examination			
	Neer test	Hawkins test	Empty can test	Drop arm test
Purpose (1)	Impingement	Impingement	Supraspinatus	Supraspinatus
Performance (4)	Proximal (scapula) stabilization Internal rotation by examiner Passive Elevation	Proximal (elbow) stabilization: Arm at 90° elevation; Elbow in 90° flexion; Forcible int. rotation of shoulder using the other hand	90° abduction of the arm, then 30° horizontal adduction (forward angling): Internal rotation (thumbs pointing toward the floor); Elbow extended; Patient resists downward pressure	Examiner abducts the patient’s shoulder to 90° Asks the patient to lower the arm to the side: Slowly Elbow extended
Positive sign (1)	Pain during passive elevation	Pain during internal rotation	Pain or weakness while resisting downward pressure	Inability to return the arm to the side slowly
Mechanism (2)	Not mentioned (0) Mentioned briefly (1) Mentioned in detail (2)			
Total	8	8	8	8

The assessors disagreed on the categorization of 22 videos (11% of the total), and consensus was reached after discussion.

### Intra- and inter-rater reliability

The intra- and inter-rater reliability of the scoring system was assessed. Two physicians evaluated all videos and used a table of random numbers to randomly select five videos per examination for re-evaluation by one researcher two weeks after the initial assessment. Intra- and inter-rater reproducibility was assessed using the weighted kappa coefficient.

### Statistical analysis

Data were analyzed with SPSS software (version 24; SPSS Inc., Chicago, IL, USA). Continuous variables were compared using one-way analysis of variance test with *post-hoc* analysis by Dunnett T3 test, and categorical variables were compared with Fisher’s exact test. Statistical significance was defined as *P*<0.05.

## Results

**Figure 1:**
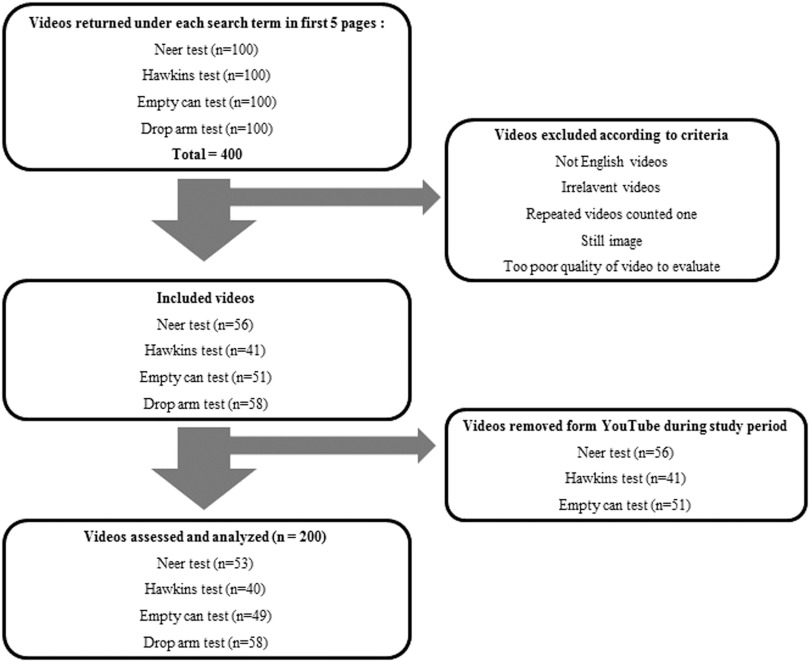
Flowchart of the selection process of videos

A total of 100 videos from the first five pages of search results for each examination were reviewed in March 2015. After applying the exclusion criteria, a total of 206 videos were identified. Because six videos became inaccessible during the study period, a total of 200 videos were finally assessed and analyzed: 53 videos for the Neer test, 40 videos for the Hawkins test, 49 videos for the empty can test, and 58 videos for the drop arm test (Figure 1).

### Usefulness of the videos

A total of 400 videos were evaluated and adopted 200 videos which yield eligible criteria. Out of 200 assessed videos, 51 (25.5%) videos were ‘very useful,’ 108 (54%) videos were ‘somewhat useful,’ and 32 (16%) videos were ‘misleading.’ Nine (4.5%) videos were categorized as ‘not useful.’

The video category was related significantly to viewers’ preferences, that is, views, views per day, and likes (Table 2). *Post-hoc* analysis (Table 3) revealed more views of ‘very useful’ videos than of ‘not useful’ and ‘misleading’ videos. ‘Somewhat useful’ videos also had more viewers than ‘not useful’ and ‘misleading’ videos. The number of views per day was generally similar, but differed significantly between the ‘very useful’ and ‘somewhat useful’ groups. The number of likes was larger for ‘very useful’ videos than for videos in the other groups, and for ‘somewhat useful’ videos compared with ‘not useful’ videos.

**Table 2:** Video demographics according to usefulness

	Usefulness				
	Very useful (6–8)	Somewhat useful (3–5)	Not useful (0–2)	Misleading	*P*-value
Videos, *n* (%)	51 (25.5)	108 (54)	9 (4.5)	32 (16)	
Days since upload (*n*)	891.8±709.9	820.3±563.3	989.2±438.5	912.0±150.4	0.629
Mean views (*n*)	36939.6±83699.4	5777.1±19001.8	712.4±1173.6	777.8±1254.8	0.002*
Mean views per day (*n*)	22.2±40.5	3.7±9.0	0.7±1.1	0.7±1.0	<0.001*
Mean ‘likes’ (*n*)	26.9±44.8	3.3±12.0	0.1±0.3	0.5±1.1	<0.001*
Mean ‘dislikes’ (*n*)	1.4±3.3	0.3±0.9	0.3±0.7	0.2±0.4	0.069

*P<0.05.

**Table 3:** *Post-hoc* analysis of video demographics according to usefulness

	Mean views	Mean views per day	Mean ‘likes’
‘Very useful’ versus ‘somewhat useful’ Adjusted *P*-value^a^	0.065	0.013*	0.003*
‘Very useful’ versus ‘not useful’ Adjusted *P*-value	0.019*	0.002*	0.001*
‘Very useful’ versus ‘misleading’ Adjusted *P*-value	0.020*	0.002*	0.001*
‘Somewhat useful’ versus ‘not useful’ Adjusted *P*-value	0.046*	0.009*	0.047*
‘Somewhat useful’ versus ‘misleading’ Adjusted *P-*value	0.045*	0.006*	0.126
‘Not useful’ versus ‘misleading’ Adjusted *P-*value	1.000	1.000	0.340

a Adjusted *P*-values were used in pairwise comparisons by Dunnett’s T3 test.

*P<0.05.

Video demographics according to uploader are shown in Table 4. The majority (67%) of videos were uploaded by individuals, whereas only 14.5% were uploaded by physicians. The numbers of views, views per day, and likes were consistently smaller for videos uploaded by individuals than for those uploaded by physicians (*P*<0.05). Video usefulness was correlated significantly with the upload source (*P*<0.001). The proportion of ‘very useful’ videos was largest among those uploaded by physicians (58.6%) and smallest among those uploaded by individuals (12.7%). Videos uploaded by individuals accounted for a larger proportion of those with misleading content (17.9%) than did videos uploaded by physicians (10.3%; Table 4). Videos were analyzed based on the physical examination, and no significant difference in usefulness was observed among examinations (*P*=0.091; Table 5).

**Table 4:** Video demographics according to uploader

	Uploader			
	Physician	Medical website	Individual	*P*-value
Videos, *n* (%)	29 (14.5)	37 (18.5)	134 (67)	
Days since upload (*n*)	1029.5±813.7	718.6±513.8	862.3±530.4	0.165
Mean views (*n*)	54627.0±102372.5	11818.9±35643.8	3481.5±11088.1	0.015*
Mean views per day (*n*)	27.9±43.8	10.6±28.1	2.5±6.0	0.004*
Mean ‘likes’ (*n*)	29.8±47.8	10.4±28.4	3.7±14.8	0.011*
Mean ‘dislikes’ (*n*)	1.6±3.0	0.9±2.9	0.2±0.8	0.025*
Usefulness, *n* (%)				<0.001**
Very useful	17 (58.6)	17 (45.9)	17 (12.7)	
Somewhat useful	8 (27.6)	14 (37.8)	86 (64.2)	
Not useful	1 (3.4)	1 (2.7)	7 (5.2)	
Misleading	3 (10.3)	5 (13.5)	24 (17.9)	

*P<0.05, physician versus individual. **P<0.05, Fisher’s exact test.

**Table 5:** Video demographics according to examination

	Examination				
	Neer test	Hawkins test	Empty can test	Drop arm test	*P*-value
Videos, *n* (%)	53	40	49	58	
Usefulness, *n* (%)					0.091
Very useful	9 (17.0)	14 (35)	15 (30.6)	13 (22.4)	
Somewhat useful	34 (64.2)	20 (50)	27 (55.1)	27 (46.6)	
Not useful	4 (7.5)	0 (0)	0 (0)	5 (8.6)	
Misleading	6 (11.3)	6 (15)	7 (14.3)	13 (22.4)	

### Intra- and inter-rater reliability

A weighted kappa score for intra-rater reliability was obtained by using 20 randomly selected videos, five for each of the four examinations (*κ*=0.847). Inter-rater reliability was calculated for each examination using a weighted kappa score; these values showed very good agreement (Neer test, *κ*=0.822; Hawkins test, *κ*=0.868; empty can test, *κ*=0.907; drop arm test, *κ*=0.854).

## Discussion

YouTube provides a large number of easily accessible videos presenting shoulder physical examinations. It could be considered an extremely helpful educational tool for shoulder physical examinations if accurate clips were selected. This study is the first attempt to assess the quality of YouTube videos as educational tools for physical examinations of the shoulder.

Shoulder pain, the third most common musculoskeletal complaint among patients visiting physicians, has a substantial impact on quality of life (Hermans *et al*., 2013). Appropriate physical examination is crucial in evaluating patients with shoulder pain because it has become a cornerstone of the diagnostic process (Hegedus *et al*., 2008). To ensure the reliability of shoulder physical examinations, precise performance of the procedures is necessary. Multimedia sources can be more helpful than conventional texts for such manual procedures, as they can provide virtual images in the 3D plane with audio descriptions.

Many studies have attempted to determine the value of YouTube videos on a variety of medical topics as informational tools for medical students (Azer, 2012; Azer *et al*., 2012; Rössler *et al*., 2012; Muhammed *et al*., 2014; Lee *et al*., 2015; Addar *et al*., 2017) and patients (Sood *et al*., 2011; Singh *et al*., 2012; Kumar *et al*., 2014; Lee *et al*., 2014; Sorensen *et al*., 2014; MacLeod *et al*., 2015; Rittberg *et al*., 2016; Kwok *et al*., 2017; Cassidy *et al*., 2018). They have yielded negative results, due mainly to the variable quality of the videos.

Only 11% of YouTube clips that dealt with laparoscopic cholecystectomy training were rated as ‘good,’ whereas 30.1% were categorized as ‘poor’ (Lee *et al*., 2015). Conversely, 27% of videos on surface anatomy (Azer, 2012), 47% of videos on neurologic examination (Azer *et al*., 2012), and 18% of videos on male urethral catheterization (Nason *et al*., 2015) were deemed useful.

Some studies have included additional information on misleading videos, which contain content that has not been proven scientifically. The information includes 16% of videos on electrocardiography (Akgun *et al*., 2014), 56% of videos on gallstone disease (Lee *et al*., 2014), and 13% of videos on lumbar puncture and spinal anesthesia (Rössler *et al*., 2012) categorized as misleading. These findings imply that searchers risk accessing misleading videos when they select YouTube videos for educational purposes without using a screening process.

In analyzing viewers’ preferences, previous studies covering clips of electrocardiography, laparoscopic cholecystectomy, femoroacetabular impingement, and pediatric adenotonsillectomy and ear tube surgery revealed no significant relationship between usefulness and viewers’ responses, such as the numbers of views, likes, and dislikes (Akgun *et al*., 2014; Sorensen *et al*., 2014; Lee *et al*., 2015; MacLeod *et al*., 2015). Moreover, in some studies pertaining to gallstone disease and hypertension, viewers displayed a preference for misleading videos (Kumar *et al*., 2014; Lee *et al*., 2014).

In the present study, however, variables reflecting viewers’ preferences, such as the numbers of views and likes, were correlated significantly with video usefulness. Viewers’ preferences were also related directly to the upload source, with greater preference found for videos uploaded by professionals and medical websites than for those uploaded by individuals. These results are in disagreement with those of a previous study of videos related to gallstone disease, which showed a lack of correlation between viewers’ reactions and uploaders (Lee *et al*., 2014).

Most (67%) videos assessed in this study were posted by individual users; physicians uploaded only 14.5% of videos. Whereas 58.6% of videos uploaded by physicians were very useful, this rate dropped to 12.7% for videos uploaded by individuals. Despite the relatively small proportion, videos uploaded by professionals had the highest quality. These results correspond to those of previous studies, which suggested that the upload source was a predictor of quality (Singh *et al*., 2012; Akgun *et al*., 2014; Lee *et al*., 2015; Madathil *et al*., 2015; Ajumobi *et al*., 2016).

In order for YouTube video to provide credibility as objectivity and accuracy, videos with a higher score could be considered to be of educational significance. Because approximately 87% of YouTube videos uploaded by individuals were included in the ‘somewhat useful,’ ‘not useful,’ or ‘misleading’ group, they were evaluated as less useful in the context of the educational value of YouTube videos. Considering that most of the videos are uploaded by individuals, it is important to select accurate videos by filtering system so that YouTube videos can be useful as educational tools.

Even those videos uploaded by the physicians may contain misleading content that can confuse novices. In the present study, ‘misleading’ videos accounted for around 1/6 of the total number of videos analyzed, and only three videos with misleading content were uploaded by physicians: one video for the empty can test and two videos for the drop arm test. Those videos also had titles that did not match the contents, raising the possibility of confusing the viewer with inappropriate knowledge of shoulder physical examinations. We also found less viewer preference for the three misleading videos uploaded by physicians than for useful videos uploaded by physicians. Some of the misleading videos uploaded by individuals and medical websites had inappropriate content, including practitioners’ demonstration of incorrect actions or ambiguous shoulder postures, and included video advertisements.

Considering our results, extra care should be taken when using YouTube clips. In this regard, filtering YouTube videos based on viewers’ preferences and the upload source can lead to the identification of reliable educational videos on shoulder physical examinations.

This study has a few limitations. First, no validated tool for the evaluation of video quality exists. We created a scoring system based on a review of the relevant literature, which was somewhat subjective and not validated. To ensure consistency, we assessed the scoring system by two physicians in the form of a checklist and obtained significant intra-rater and inter-rater agreement. Two reviewers would not be enough to prove the reliability. Second, this study was performed in a cross-sectional manner. The exclusion of six videos because of their disappearance during the study period highlights the temporary character of YouTube. YouTube is a dynamic repository of video information and search results may vary over time (Lee *et al*., 2014). In addition, our analysis was limited to content located by direct searches on the YouTube site, and the results may not apply to videos accessed from directed links on other websites. And, non-English-language videos were excluded from the analysis.

In this study, significant relationships between video usefulness and viewers’ preferences were revealed. Video usefulness was also correlated with the upload source, with higher-quality content seen among clips uploaded by official hospitals and university-affiliated organizations.

YouTube could be used as an effective informational resource if an appropriate selection process is applied. Review of the upload source and viewers’ responses could help to identify higher-quality videos on shoulder physical examinations.
